# Hyperthermic Intraperitoneal Chemotherapy in the Management of Gastric Cancer: A Narrative Review

**DOI:** 10.3390/ijerph19020681

**Published:** 2022-01-07

**Authors:** Marek Mazurek, Małgorzata Szlendak, Alicja Forma, Jacek Baj, Ryszard Maciejewski, Giandomenico Roviello, Luigi Marano, Franco Roviello, Karol Polom, Robert Sitarz

**Affiliations:** 1 Department of Surgical Oncology, Voivodship Hospital in Siedlce, 08-110 Siedlce, Poland; marekmazurek1@o2.pl; 2 Department of Human Anatomy, Medical University of Lublin, 20-090 Lublin, Poland; malgorzata.skierucha@gmail.com (M.S.); jacek.baj@umlub.pl (J.B.); maciejewski.r@gmail.com (R.M.); 3 Department of Oncology, Medical University of Warsaw, 02-097 Warsaw, Poland; 4 Department of Forensic Medicine, Medical University of Lublin, 20-090 Lublin, Poland; aforma@onet.pl; 5 Department of Health Sciences, University of Florence, 50139 Florence, Italy; giandomenico.roviello@unifi.it; 6 Department of General Surgery and Surgical Oncology, University of Siena, 53100 Siena, Italy; luigi.marano@unisi.it (L.M.); franco.roviello@unisi.it (F.R.); 7 Department of Surgical Oncology, Medical University of Gdansk, 80-070 Gdansk, Poland; polom.karol@gmail.com; 8 Department of Surgical Oncology, St. John’s Cancer Center, 20-090 Lublin, Poland

**Keywords:** gastric cancer, hyperthermic intraperitoneal chemotherapy, peritoneal metastasis, cytoreductive surgery, cancer treatment

## Abstract

Gastric cancer (GC) patients with peritoneal metastasis tend to achieve poor clinical outcomes. Until recently, the treatment options were limited mainly to either palliative chemotherapy or radiation therapy in exceptional cases. Currently, these patients benefit from multimodal treatment, such as cytoreductive surgery (CRS) with hyperthermic intraperitoneal chemotherapy (HIPEC). Despite good overall results, this treatment modality is still widely debated. The following study is designed to assess the papers about the possible application and utility of HIPEC in GC. A search in the PubMed, Web of Science, and Scopus databases was performed to assess the papers devoted to the role of HIPEC in GC treatment; a literature search was performed until March 21st; and, finally, 50 studies with a total number of 3946 patients were analyzed. According to the most recent data, it seems to be reasonable to limit the duration of HIPEC to the shortest effective time. Moreover, the drugs used in HIPEC need to have equal concentrations and the same solvent. Perioperative chemotherapy needs to be reported in detail and, furthermore, the term “morbidity” should be defined more clearly by the authors.

## 1. Introduction

Hyperthermic intraperitoneal chemotherapy (HIPEC) is a surgical procedure that aims to deliver heated chemotherapy directly to the abdomen after surgery; the procedure was invented and firstly used more than 20 years ago [1]. Currently, HIPEC is an emerging procedure aimed to treat peritoneal metastasis of gastric cancer (GC); unfortunately, data about HIPEC application in locally advanced GC is still scarce. This treatment modality is considered to provide beneficial results in the management of several clinical syndromes within the peritoneum, such as peritoneal mesothelioma, pseudomyxoma peritonei, and peritoneal metastasis (PM), as a result of the metastatic properties mainly of several cancers, including colorectal, ovarian, or gastric cancer [2,3,4,5,6,7]. However, in GC patients, HIPEC is still considered as an innovative way to both prevent and treat PM, which is diagnosed in approximately 30% of patients with advanced GC. PM is characterized by a very poor prognosis; even though it is just a regional condition restricted only to the peritoneum, it is usually fatal with a maximal 3-month-long prognosis surgery (CRS) in a natural course, prolonged operation, as well as great intraoperative hemorrhages [8,9].

**Figure 1:**
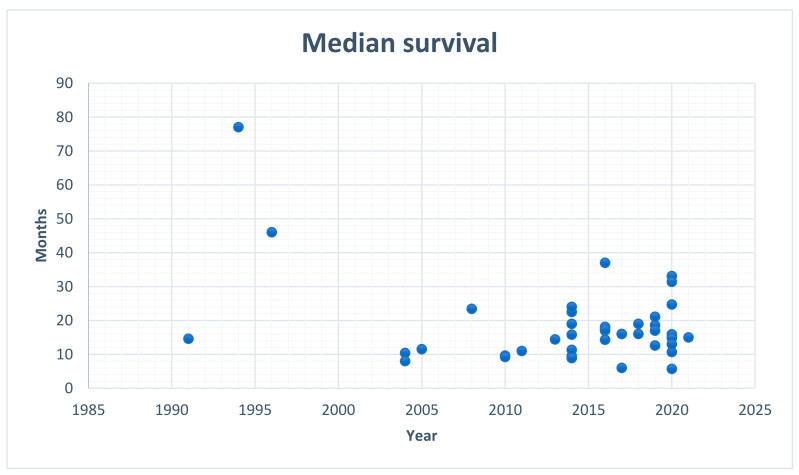
Median survival in patients with gastric cancer treated with HIPEC; data from the analyzed papers.

HIPEC, as a potential treatment modality for GC patients, is not included as a part of the current national guidelines, despite reported effectiveness as well as long-term survival rates. Researchers consistently question the potential benefits of HIPEC in terms of GC treatment and, so far, they have tested many modalities of the procedure over time. Granieri et al., in their meta-analysis of randomized controlled trials (2021), reported that a combination of CRS with HIPEC seems to be beneficial for patients with locally advanced GC, in prophylactic as well as curative settings [10]. Despite the extensive experience and the multitude of studies, along with the usage of HIPEC in the leading oncological centers all over the world, there are still no direct recommendations regarding its application as a treatment modality in GC patients. Moreover, it appears that, since the first attempts of HIPEC in GC, the median survival, which is the most important parameter, has not significantly changed (Figure 1).

This observation leads to a further discussion on the possible modifications that can be implemented to improve the utility of this procedure, and this should be primarily based on the results of the independent clinical studies. One of the most prevalent conundra regarding HIPEC therapy worldwide, includes the not yet established proper doses of drugs intraperitoneally administered, which is currently non-evidence-based due to the lack of proper recommendations [11]. However, even a brief analysis of the reports devoted to the application of HIPEC in GC patients shows that they present very difficult material for a comparative analysis, due to a wide spectrum of methodological differences applied in those studies. In order to draw credible and clinically useful conclusions, clinical trials need to be comparably reported. Moreover, the role of HIPEC in the treatment of PM in GC is still evolving and continually modified at all stages of treatment. Firstly, it is more frequently used in neoadjuvant therapy, such as neoadjuvant laparoscopic, heated intraperitoneal chemotherapy (NLHIPEC) after systemic chemotherapy, or neoadjuvant intraperitoneal and systemic chemotherapy (NIPS) in a bidirectional manner (BIPSC), used for both the intraperitoneal (IP) and intravenous (IV) routes of chemotherapy administration before the CRS. Secondly, it is used in the prophylactic treatment (P-HIPEC) and palliative treatment to minimize the risk and reduce the number of the ascites [2,12,13,14,15]. Apart from HIPEC, other forms of intraperitoneal chemotherapy, such as the early postoperative chemotherapy (EPIC) along with normothermic intra-operative intraperitoneal chemotherapy (NIIC), normothermic intraperitoneal chemotherapy long-term (NIPEC-LT), repeated intraperitoneal chemotherapy (RIPEC) [13,15,16,17], or pressurized intraperitoneal aerosol chemotherapy (PIPAC), with the relative benefits of delivering aerosolized chemotherapy under pressure into the abdominal cavity, are more frequently used [12,13,16].

The present study assesses the papers about the possible application of HIPEC in the treatment of GC patients, including those with GC and concomitant PM, with regard to the details, guidelines, and recommendations described by the researchers in the studies chosen for this review. Furthermore, we summarize the current state of knowledge regarding the discrepancies of the HIPEC technique applied in GC patients, with an emphasis on the inaccuracies concerning the technique duration as well as the agents, doses, and solvents used in different medical centers. This review will provide an insight into a broad spectrum of potential modifications regarding HIPEC itself, enabling a further exploration of this technique and its possible standardization for a specific group of GC patients who would potentially benefit from this technique.

## 2. Methods and Materials

### 2.1. Search Strategy

The PubMed, Web of Science, and Scopus databases were searched to assess the papers devoted to the role of HIPEC in the treatment of GC with a particular emphasis on the patients with PM present. The search string was as follows: “(gastric cancer) AND (HIPEC) OR (hyperthermic intraperitoneal chemotherapy) AND (peritoneal metastasis)”. The time period was restricted to 1 August 1989 and 21 March 2021 (32 years). The search was only restricted to the English language. During the first identification, which was the primary research conducted in March 2021, a total number of 552 papers was retrieved. After the removal of the duplicates, a total of 236 articles was included in the first analysis. Due to the disqualification of case reports, comments to other papers, letters to Editors, and papers devoted to other topics, a total number of 143 papers were assessed for eligibility. Due to the inaccessibility of several papers, and not considering the articles devoted to the tumors other than GC, the final analysis was based on a total number of 50 papers (Appendix A).

In every studied article, information was sought based on the following data: the number of patients; their medium age; agents and doses used in the HIPEC treatment of GC patients with or without PM; volume and kind of solvent for perfusate; duration of HIPEC; the modality of the procedure (open/closed); information about perioperative chemotherapy; the value of mortality and morbidity and their definition and interpretation, according to the authors of the paper; a detailed list of complications; as well as the median survival rates. The results are presented in Table A1 (Appendix A).

### 2.2. Details of the Studied Population

The analyzed reports varied with regards to the population of patients. The largest study included 249 patients, while the smallest was restricted to only 9 patients. The age range of patients was between 47 and 61 years; however, at least 5 papers did not mention this parameter at all, which is one of the limitations of this study.

## 3. Results

### 3.1. Intraperitoneal Chemotherapy

Intraperitoneal chemotherapy provides significantly higher local concentrations, which results in a direct anti-tumor effect on free peritoneal cancer cells. Therefore, intraperitoneal chemotherapy facilitates its uptake by cancer cells, by the enhanced drug penetration into them. In terms of the drugs used in HIPEC, mainly four variables differed in the analyzed reports: agents, doses, kind, and volume of the solvents. The most common chemotherapeutic agents used were Mitomycin C and Cisplatin, which were applied in 31 and 32 regimens, respectively. The doses of the applied agents were reported either as the whole dose for the procedure in milligrams (mg) (19 papers) [18,19,20,21,22,23,24,25,26,27,28,29,30,31,32,33,34,35,36], or in milligrams for the body surface area (mg/m^2^) (26 papers) [11,35,37,38,39,40,41,42,43,44,45,46,47,48,49,50,51,52,53,54,55,56,57,58,59,60]. One of the latter units was additionally expressed in terms of the volume of solution (mg/m^2^/L) [48]. Two authors did not report the doses of the drugs [61,62,63]; in older studies, the doses were once reported in either µq/mL [64] or once in mg/kg [65]. Several articles lacked information about the volume of perfusate; those were mainly newer reports. In most of the cases, drugs were administered in the saline solution; however, there were single cases where the saline was replaced with 5% dextrose in water (D5W) or dialysis solution.

### 3.2. Duration of HIPEC

In the studied reports, there was insufficient information about the duration of the HIPEC procedure. Generally, the procedure lasted for approximately 60 or 90 min [18,19,20,21,23,24,25,26,29,32,33,35,36,37,38,42,43,44,46,47,48,49,50,51,52,57,58,59,61,64,65,66]. De Roover et al. reported that, in half of their cases (*n* = 8), there was a need to shorten the duration of HIPEC due to central hyperthermia [37]. Some researchers (mainly in newer reports) shortened the HIPEC duration to 30 or 45 min [11,45,53,54,55,56,67]. The others did not seem to value and stick to the time frames, and applied HIPEC for a time frame that was not directly specified; they preferred to choose a time range somewhere within the 30–120 min time frame [22,27,28,30,31,34,39,40,41,60,62,63].

### 3.3. Perioperative Systemic Chemotherapy

Most of the analyzed papers presented very modest information about the chemotherapy administered before and following the CRS + HIPEC. In 32 of the analyzed articles [11,18,19,20,21,22,23,30,31,34,36,38,39,40,41,42,43,44,45,46,47,50,51,52,54,55,58,60,61,62,63,66], the issue of the perioperative systemic treatment was at least mentioned (in the last analysis, the authors perceived it as an important factor), and in 19 papers it was not discussed at all [24,25,26,27,28,29,32,33,35,37,48,49,53,56,57,59,64,65,67].

### 3.4. Mortality

Ultimately, the analysis showed that mortality due to the application of HIPEC in GC patients is low. Only in 7 of the analyzed papers, the mortality rate exceeded 6%, but in 4 of them, these results were associated with a small number of the studied population (*n* = 9, 12, 16, and 17 respectively) [19,37,39,45,48,60,66]; only one postoperative death was reported. In fifteen of the analyzed papers, the mortality rate was 0% [11,22,24,25,26,28,30,38,44,46,49,51,52,63,65]. 

### 3.5. Morbidity

In the majority of cases, the term “morbidity” was defined as the occurrence of major postoperative complications, and it was reported in a wide range of percentages (from 5.6% to 72%). In the analyzed papers, some authors precisely listed the morbid events related to treatment or even made a brief comment (27 articles) [21,22,23,24,25,27,29,30,32,34,35,38,42,44,45,46,47,50,51,54,55,56,57,58,60,61,65], the others reported only numbers (13 articles) [11,18,19,20,26,28,39,40,41,48,49,52,62], and, in several papers, the morbidity was not discussed in the results (11 articles) [31,33,36,37,43,53,59,63,64,66,67]. In three papers, the complications were divided into surgery and HIPEC-related.

### 3.6. Median Survival

In some analyses, the patients were divided into the following groups: 1) curative, adjuvant, palliative, 2) patients who underwent CRS and HIPEC or only CRS, 3) patients with PCI < 6, and PCI > 6, and 4) complete cytoreduction, or not complete (Appendix A).

## 4. Discussion 

HIPEC is a treatment strategy that, combined with surgery, aims to treat advanced cancers within the abdomen, such as colorectal cancer, gastric cancer, ovarian cancer, or peritoneal mesothelioma. Even though its usefulness was reported in several types of advanced cancers within the abdomen [68,69,70], it should be considered that the application of HIPEC is associated with a risk of several complications, including hematological toxicity, kidney failure, venous thromboembolism, and infections within the venous accesses and urinary tract [71]. Typical side effects include nausea, vomiting, fatigue, or weight loss, but those usually persist up to 3 months after surgery. The other most common complications include fatigue, disturbed sleep pattern, bloating, diarrhea, or constipation; depression is also reported as a side effect. Generally, the occurrence of any adverse events reflects the risks of the whole operation, but distinguishing between surgical and systemic complications can be additionally relevant in further analysis concerning the safest combination of agents and their doses. However, recently, the contemporary safety of HIPEC has significantly improved. Even though the morbidity and mortality rates remain relatively high in both HIPEC and CRS, the associated learning curve is steep and numerous well-structured tutor-based training programs have so far been implemented in Europe, to progressively overcome those drawbacks [72].

The authors of the reports that were directly devoted to HIPEC, usually discussed PM as an independent diagnosis, which, regardless of the origin, has a similar course and prognosis; therefore, it should be treated with the same means. According to the papers included in this narrative review, there are no clear indications as well as recommendations regarding the details of HIPEC, such as the types of agents and solvents (as well as their doses) used, along with the time of HIPEC duration. There are also discrepancies concerning the perioperative systemic chemotherapy applied in GC patients. For these reasons, it can be assumed that even though HIPEC seems to be beneficial for some of the GC patients, this type of therapy should be evaluated further and more standardized amongst the clinical centers; the group of patients who would be most beneficial to this therapy should also be investigated. In Poland, so far, there are only eight medical centers in which HIPEC is applied in GC patients; however, due to a high number of inconsistent data regarding the HIPEC procedure itself, there is no publication on this subject yet.

In our paper, 41 reports were excluded from further analysis, although they contained the required information about HIPEC in GC patients with PM. The reason was that the information about the patients’ characteristics (age and profile of chemotherapy, as well as outcomes data), rates of mortality, morbidity, and median survival, were reported together for the patients with PM from tumors of other origins.

Regarding the role of HIPEC in peritoneal carcinomatosis, more randomized trials still need to be conducted in order to select those patients who would constitute good candidates for such therapeutic approaches. In addition to the clinical features, the molecular and pathological features should be investigated in order to select patients for whom HIPEC would be beneficial. Apart from gastric cancer, minimally invasive secondary cytoreduction combined with HIPEC is currently under investigation to be applied in other peritoneal cancers, especially in the case of patients with ovarian cancer [73,74].

### 4.1. Intraperitoneal Chemotherapy

The diversity of regimens used for HIPEC in patients with GC and PM is understandable as, for the time being, the role of this treatment modality is still unsettled and there are no strict restrictions for this matter. The most common chemotherapeutic drugs used in HIPEC include Mitomycin-C and Cisplatin [75]—there were also reports about the potential application of oxaliplatin and doxorubicin; however, those are less common drugs [13]. However, the variability in the reports about the doses of the drugs and the lack of designation of the volume of perfusate during planning procedures, resulted in the situation that, even in the same institutions, the patients are treated with solutions of different concentrations of the drugs. It seems that the rules of treatment with HIPEC can be similar to dialysis therapy. Treating patients incomparably implies that the solutions of the drugs used during treatment need to have equal concentrations, expressed in the unit of mass to the volume of the solution [mg/mL]. The solvent needs to be universal—a saline solution, as in the majority of cases. Then, the volume of the applied drug solution should depend on the body surface area of the patient. 

### 4.2. Duration of HIPEC

The duration of HIPEC depends on the used protocol and significantly varies depending on the type of chemotherapeutic agent used along with its pharmacokinetics features. One of the questions that have appeared in this study is whether the duration accuracy of the applied procedure matters. From the pharmacokinetic point of view, the depth of drug penetration is very minimal (up to 1–3 mm), and the prolongation of the treatment time would not increase this [76]. On the other hand, hyperthermia increases blood circulation and the longer the drugs solution remains in the peritoneal cavity, the more the drugs are prone to penetrate the vessels and enter into the blood. However, even if this course of events occurs, it is very limited, as the concentration of drugs in the blood after the application of HIPEC is far below the toxicity threshold [65]. However, the real problem exists from surgical and anesthesiological points of view, as a prolonged operation course increases the rate of postoperative morbidity, blood loss, as well as infection risk [8]. Therefore, some studies suggest that it would be reasonable to limit the duration of HIPEC to the shortest effective time, which means that the desired effects (favorable clinical outcomes with a minimization of the above-mentioned side effects) would be performed in the shortest time possible, without the additional risk of potential intraoperative side effects (either surgical or anesthesia). 

### 4.3. Perioperative Chemotherapy

Perioperative chemotherapy (high rates of systemic chemotherapy in the neoadjuvant and adjuvant settings) is a favorable prognostic factor with a positive effect on survival [2,18,19,38]. The aim of this report was not to analyze the details of perioperative chemotherapy, but rather to assess the frequency of reporting this parameter in studies. Based on our analysis, it appears that this issue is generally neglected. In only 17 papers, there was some information about the types of agents used, doses, schedule of regimens, several patients qualified for chemotherapy, and reasons of disqualification; however, in very few, the data was complex. The probable reason is that the analyzed reports were mainly addressed to the surgeons and too much data can lead to information noise. However, from a multidisciplinary point of view, the factor of perioperative chemotherapy is relevant, as it diversifies the treated population. 

### 4.4. Mortality

HIPEC is always the complement to severe and extensive CRS and; therefore, “the mortality rate” must be considered throughout the procedure. In most of the studied articles, “mortality” was defined as the number of deaths in 30 postoperative days. However, sometimes the authors redefined this entry and extended the mortality-free period to emphasize the safety of the procedure. In the literature, the careful and rigorous selection of patients qualified for CRS and HIPEC was often underlined as the crucial factor contributing to the effectiveness of treatment [2,38,77].

### 4.5. Morbidity

While “mortality” is an easy and unambiguous event to be defined, and the term “morbidity” is interpreted variously by different authors. It seems that one of the best options is to grade the adverse events related to CRS and HIPEC, according to the “common terminology criteria for adverse events” valid for the time of publication [78]. However, the authors did not always consider the same grades in the final analysis. Most often, they reported grade III–IV, but, in some papers, the less severe grades along with the fatal grade V were also included. Eventually, when analyzing a single paper, the way of reporting a matter of morbidity seemed satisfactory; however, in a wider perspective, the percentage numbers were very misleading, as they could not be compared. Complications are usually graded using the Clavien–Dindo classification system [79]. CRS combined with HIPEC presents significantly lower mortality and morbidity rates, compared with other major gastrointestinal surgical procedures [80]. 

### 4.6. Median Survival

The estimated median survival rate of patients with GC and concomitant PM is about 6–18 months [81]. Of the analyzed papers, the lowest median survival was mentioned in a study [28] by Hall et al. (2004), while the highest median survival rate was reported by Hamazoe et al. (1994) [64] with the values being equal to 8 months and 77 months, respectively. The appropriate selection of patients using the Peritoneal Carcinomatosis Index (PCI) < 6 and complete cytoreduction, showed promising results in improving overall survival (OS) rates [12,16,82]. The implementation of HIPEC in the case of patients with GC and PM seems to be reasonable, since, even though the median survival rates differ among single-center or prospective registry studies, they are continually improving, not only due to the favorable modifications of the technique itself, but also because of proper surgical training [72].

## 5. Conclusions

According to the studies devoted to the application of HIPEC in GC patients, it seems that at least the selected patients can benefit from this type of therapy. Even though similar, the technique itself is continually modified and differs between clinics in terms of the solvents and agents used, as well as the duration of the whole procedure. Further studies, with long-term evaluations, are of major importance to identify the prognostic factors that either positively or negatively affect the overall survival rate of GC patients treated with HIPEC. Numerous studies regarding this matter are currently ongoing, as researchers worldwide try to investigate those factors; e.g., Graziosi et al. indicate the patient-related parameters (pre-operative serum albumin level or platelets-to-lymphocytes ratio) as well as the tumor-related factors (such as the primary tumor site or PCI) as factors strongly associated with the survival of operated patients [83]. Patients with low CC scores present a significant survival advantage [10]. What is crucial, while considering the outcome of HIPEC, is the proper patient selection. Cocollini et al. suggest that the morbidity rate of patients is incremented by intraperitoneal chemotherapy [84]. In the papers devoted to HIPEC in the treatment of PM of any origin, it would be beneficial to distinguish the detailed data of patients and results of the procedures for populations of the same primary neoplasm. To draw credible conclusions and finally settle the role of HIPEC in GC, the reports need to fulfill several conditions:

- Solutions of the drugs used in HIPEC need to have equal concentrations, expressed in the unit of mass to the volume of solution [mg/mL]; the solvent needs to be universal, and the volume of the solution should depend on the body surface area of the patient, as well as the optimal doses of intraperitoneal administrated chemotherapy agents’ doses.
- The information about perioperative chemotherapy needs to be reported and should contain details about the chemotherapeutic agents used, their doses, the schedule of regimens, how many patients qualified for chemotherapy, and the reasons for disqualification.

The term “morbidity” should be clearly defined. We suggest applying to the observations in the majority of reports the consideration of “morbidity” as grade III–IV adverse events that comes from too common terminology criteria for adverse events [78]. Moreover, it is not enough to specify the number; however, morbid events should be listed. Currently, several randomized clinical trials (RCTs) have been conducted; however, with quite different conclusions regarding the usage of CRS and HIPEC in patients with gastric cancer. The Italian Association of Medical Oncology (AIOM) strongly advises against the application of CRS and HIPEC in patients with PM, while the Peritoneal Surface Oncology Group International (PSOGI) suggests that patients with gastric cancer and PM can strongly benefit from such treatment [85,86]. Such discrepancies once again suggest that RCT, as well as seeking a potential standardization, are of a major necessity.

## Abbreviations

CDD	cisplatin
CRS	cytoreductive surgery
EPIC	early post-operative chemotherapy
GC	gastric cancer
HIPEC	hyperthermic intraperitoneal chemotherapy
IP	intraperitoneal
IV	intravenous
LDG	laparoscopy distal gastrectomy
NIIC	normothermic intra-operative intraperitoneal chemotherapy
NIPEC-LT	normothermic intraperitoneal chemotherapy long-term
NIPS	neoadjuvant intraperitoneal and systemic chemotherapy
NLHIPEC	neoadjuvant laparoscopic, heated intraperitoneal chemotherapy
MMC	mitomycin C
PM	peritoneal metastasis
P-HIPEC	prophylactic heated intraperitoneal chemotherapy
PIPAC	pressurized intraperitoneal aerosol chemotherapy
RIPEC	repeated intraperitoneal chemotherapy

## Appendix A

**Table A1:** Content of the analyzed papers.

Ref.	Authors	Year	No. Patients	Mean Age (Years)	Agent	Dose	Solvent	Time	Technique	Perioperative Chemotherapy	Mortality	Morbidity	Median Survival
[53]	Zhu et al.	2020	43 (22 CHIP treatment, 21 chemotherapy)	51.0 (CHIP group) 55.0 (chemotherapy group)	CDDP	75 mg/m^2^	Saline	30	ND	ND	ND	ND definition—complications listed	Not reached (CHIP group); 33.1 months (group with chemotherapy alone)
[54,55]	Koemans et al.	2021	25 (gastrectomy, CRS, HIPEC)	60.0	Oxaliplatin	460 mg/m^2^	ND	30 min 90 min	ND	Yes	ND	Serious adverse events—68.0%	15 months
					Docetaxel	0, 50, 75 mg/m^2^ (escalating doses)							
[35]	Ji et al.	2020	125 (CRS + HIPEC)	51.0	CDDP, MMC (CDDP + MMC)	120 mg (CDDP) 30 mg (MMC)	Saline	60 min or 90 min	ND	ND	30 day perioperative mortality—0.9%; 90 day postoperative mortality—3.2%	Serious adverse events—8.8%	10.7 months; 1 year—43.8%, 2 years—24.7%, 3 years—18.6%, 5 years—15.7%
					CDDP, Docetaxel (CDDP + DOC)	120 mg 120 mg							
					Lobaplatin, Docetaxel (LP + DOC)	50 mg/m^2^ 60 mg/m^2^							
[36]	Blumenthaler et al.	2020	52 (25 LS-HIPEC, 27 standard care (SC))	57 (LS-HIPEC group), 64 (SC group)	MMC, CDDP	30 mg (MMC) 200 mg (CDDP)	ND	60 min	Closed	Yes	ND	ND	24.7 months (LS-HIPEC group), 21.3 months (SC group); 1 year—95.5% (LS-HIPEC group), 76.9% (SC group); 2 years—57.2% (LS-HIPEC group), 19.1% (SC group); 3 years—19.1% (LS-HIPEC group), 9.6% (SC group)
[56]	Yin et al.	2021	138 (92 LDG , 46 LDG + HIPEC)	53.3 (LDG group), 52.5 (LDG + HIPEC group)	CDDP	75 mg/m^2^	6 L of heated saline	45 min	Closed	ND	ND	Complications: 11.96% (LDG group), 13.04% (LDG + HIPEC group) Abdominal recurrence after 2 years from operation: 10.87% (LDG group), 4.35% (LDG + HIPEC group)	ND
[67]	Fan et al.	2021	50 (33 HIPEC with CDDP, 17 adjuvant chemotherapy with SOX regime)	61.0	CDDP	50 mg/L	0.9% sodium chloride	30 min	ND	ND	ND	ND definition-Complications listed	3 years—92.0% (87.9% in HIPEC group, 100% in adjuvant chemotherapy group)
[57]	Rosa et al.	2021	85 (39 CRS, 23 gastrectomy + curative HIPEC, 23 gastrectomy + prophylactic HIPEC)	61.0 (68 CRS, 52 gastrectomy + curative HIPEC, 58 gastrectomy + prophylactic HIPEC)	MMC, CDDP	15 mg/m^2^ (MMC) 75 mg/m^2^ (CDDP)	2 L/m^2^ 0.9% NaCl solution	90 min	Open	ND	Death 30 days from surgery—5% (CRS), 4% (gastrectomy + curative HIPEC), 0% (gastrectomy + prophylactic HIPEC)	Postoperative complications within 30 days from surgery—46% (CRS), 39% (gastrectomy + curative HIPEC), 39% (gastrectomy + prophylactic HIPEC)	5 years—9% (CRS), 27% (gastrectomy + curative HIPEC), 33% (gastrectomy + prophylactic HIPEC)
[66]	Xie et al.	2020	113 (51 HIPEC + adjuvant chemotherapy, 62 adjuvant chemotherapy)	60.9 (HIPEC + adjuvant chemotherapy), 61.5 (adjuvant chemotherapy)	CDDP	50 mg/L	saline	60 min	Open	No	17.6% (HIPEC group), 38.7% (conventional adjuvant chemotherapy group)	ND definition—complications listed	1 year—96.1% (HIPEC group), 95.2% (conventional adjuvant chemotherapy group). 3 years—68.6% (HIPEC group), 66.3% (conventional adjuvant chemotherapy group)
[58]	Yu et al.	2020	38 (18: neoadjuvant systemic chemotherapy + HIPEC + CRS; 20: chemotherapy + HIPEC)	52.0 (49.8 neoadjuvant systemic chemotherapy + HIPEC + CRS; 53.5: chemotherapy + HIPEC)	Paclitaxel	75 mg/m^2^	3 L of heated 0.9% saline	60 min	ND	Yes	ND	28.9% adverse events (grade 3 or 4)	15.1 months (21.1 months (neoadjuvant systemic chemotherapy + HIPEC + CRS), 10.8 months (chemotherapy + HIPEC))
[59]	Lei et al.	2020	498 (249 HIPEC + chemotherapy, 249 chemotherapy)	55.3 (54.6 HIPEC + chemotherapy, 56.0 chemotherapy)	Paclitaxel or platinum (oxaliplatin: or CDDP)	75−100 mg/m^2^ 100−130 mg/m^2^ 50−75 mg/m^2^	ND	60 min	Closed	ND	ND	ND definition—complications listed	15.9 months (HIPEC + chemotherapy), 10.8 months (chemotherapy)
[63]	White et al.	2020	70 (LS-HIPEC)	54.3	43 patients (MMC + CDDP)	ND	ND	ND	ND	Yes	Death 30 days after LS-HIPEC—0% Death 30 days after LS-HIPEC—4%	ND	31.4 months (patients without gross carcinomatosis PCI = 0); 14.8 months (patients with PCI scores of 1–7); 5.7 months (patients with PCI > 7)
				55.6	27 patients (MMC + CDDP + Paclitaxel)								
[39]	Bonnot et al.	2019	277 (180 CRS-HIPEC, 90 CRSa)	59.8 (CRS-HIPEC), 51.1 (CRSa)	Monochemotherapy (77.2%) MMC CDDP Oxaliplatin 22,8 % drug combination	30–50 mg/m^2^ 50–100 mg/m^2^ 300–460 mg/m^2^	ND	30–120 min	Open (40.6%), closed (59.4%)	Yes	3.2% (30 days), 8.4% (90 days)	54.3 %, 53.7% (CRS-HIPEC), 55.3% (CRSa)	18.6 months (CRS-HIPEC), 11.4 months (CRSa)
[11]	Hotopp et al.	2019	26	50	Taxotere	80 mg/m^2^	4 L rinse solution	45 min	Open	Yes	0% (30 days)	26.9%	17 months
					Oxaliplatin	200 mg/m^2^							
[40]	Yarema et al.	2019	117	54.1	MMC + CDDP	12.5 mg/m^2^ + 75 mg/m^2^ 460 mg/m^2^		30–90 min	Open	Yes	5.1%	29.1%	12.6 months survivals 1 year—53.8% (curative group); 34 months survival 1 year—91.7% (adjuvant group); 3.5 months survival 1 year—0% (palliative group)
					Oxaliplatin								
					MMC	10–15 mg/m^2^							
					CDDP + Doxorubicin	75 mg/m^2^ + 15 mg/m^2^							
					CDDP	75 mg/m^2^							
[41]	Rau et al.	2020	235	53.4	CDDP, Doxorubicin, MMC, Oxaliplatin with combination CDDP + DoX, CDDP + MMC	75 mg/m^2^, 15 mg/m^2^, 30 mg/m^2^, 300 mg/m^2^	ND	30–90 min	Closed (184), open (51)	Yes	5.1%	17,0%	13 months
[62]	Manzanedo et al.	2019	88	53	CDDP +Doxorubicin, MMC + CDDP, MMC, Oxaliplatin	ND	ND	ND	Open (63) closed withCO^2^ (22)	Yes	3.4%	31,0%	21.2 months survivals 1 year—79.9%; 3 year—30.9%; and 5 year—27.5%
[42]	Rihuete et al.	2018	35	53	CDDP	100 mg/m^2^	ND	90 min	Open	Yes	5.7% (90 day)	25.7% serious adverse events (grade IIIb–V)	16 months survivals 1 year—70.8%; 3 year—21.3%; 5 year—21.3%
					Doxorubicin	15 mg/m^2^							
[18]	Kim et al.	2018	38	45.8	MMC + CDDP	30 mg 90 mg	ND	90 min	Closed	Yes	5.7%	42.1%	19 months
[38]	Topal et al.	2017	32	58	CDDP	100 mg/m^2^	3–4 L saline	60 min	Open	Yes	0%	72%—postoperative including 16%—nephrotoxity	16 months survivals 1 year—71.9%; 3 year—14.1%; 5 year—3.5%
[19]	Fugazzolaet al.	2017	17	53	CDDP + Paclitaxel	(150.3 mg -175.9 mg) + (263 - 302.8 mg)		90 min	Open	Yes	8%S PC, 50% MPC	61% SPC, 100 % MPC	16 months (SPC), 6 months (MPC)
					MMC + CDDP	(26–27.5 mg) + (163–173 mg)—mean dosage							
[20]	Geng et al.	2016	312 (40 HIPEC)	53,9	Docetaxel	120 mg	3.5 L normal saline	60 min	Closed	Yes	ND	11,2%	17 months
[21]	Tu et al.	2016	231	55.1	5FU	1500 mg	4.5L ofsaline	60 min	Closed	Yes	0.9% postoperative	6.9% postoperative (grade I–IV)—complications listed	37 months
					CDDP	100 mg							
[43]	Boerner et al.	2016	38	52.6	CDDP	75 mg/m^2^	ND	60 min	Closed	Yes	ND	ND	18.1 months
					Doxorubicin	15 mg/m^2^							
[44]	Wu et al.	2016	50	ND	Lobaplatin	50 mg/m^2^	6 L of saline	60 min	Open	Yes	0% postoperative	23.1%* postoperative 30 days (grade III–V)	14.3 months
					Docetaxel	60 mg/m^2^							
[61]	Chia et al.	2016	81	ND	MMC CDDP/OX	ND ND	ND	90 min	Closed/ Open	Yes	2.5% 30 days	44% postoperative (grade III–IV)	17.3 months
[22]	Magge et al.	2014	23	51.5	MMC	40 mg	Saline	100 min	Open	Yes	0 % 60 days	52.2% ND period (grade III–IV)	9.5 months
[45]	Rudloff et al.	2014	9	ND	OX i.v. 5FU + leucovorin	460 mg/m^2^ 400 mg/m^2^	5% dextrose in water (D5W)	30 min	Open	Yes	11.1% 90 days	90 days (grade III–V)	11.3 months
[46]	Königsrainer et al.	2014	18	56	CDDP	50 mg/m^2^	ND	90 min	Open	Yes	0% 30 days	46% ND period (grade I–IV)	8.9 months
[47]	Yarema et al.	2014	49	ND	MMC	12.5 mg/m^2^	ND	90 min	Closed	Yes	4.1% postoperative	26.5% postoperative (grade III–IV)—distinguished between surgical and HIPEC	22.5 months
					CDDP	75 mg/m^2^							
[48]	Saladino et al.	2014	12	ND	MMC	25 mg/m^2^/L	ND	90 min	Closed	ND	8.3% ND ND	33.3% ND ND	24 months
					CDDP	3.3 mg/m^2^/L							
[49]	Muller et al.	2014	26	53	OX	200 mg/m^2^	ND	90 min	Closed	ND	0% 30 days	23% postoperative ND definition	19 months
					Docetaxel	80 mg/m^2^							
[50]	Canbay et al.	2014	152	51.5	Docetaxel	30 mg/m^2^	ND	90 min	Open	Yes	3.9% postoperative 30 days	23% perioperative (grade I–V)—complications listed	15.8 months
[23]	Glehen et al.	2004	49	53,7	MMC	40–60 mg	4–6 L	90 min	Closed	Yes	4% 30 days	27% postoperative 30 days ND definition—complications listed	10.4 months
[51]	Hultman et al.	2013	8	ND	Doxorubicin	15 mg/m^2^	ND	90 min	Open	Yes	0% postoperative 30 days	62.5% perioperative (grade II–IV)	14.4 months
					CDDP	50 mg/m^2^							
				ND	(3 patients) OX i.v. 5FU Leucovorin	460 mg/m^2^ 500 mg/m^2^ 60 mg/m^2^		30 min				Complications listed	
[24]	Mizumoto et al.	2012	13	48	MMC	20 mg	Saline	60 min	Open	ND	0% 30 days	19% 38% postoperative (grade I–II and III–IV)	ND
					CDDP	1000 g							
[52]	Costa et al.	2012	10	47	MMC	34 mg/m^2^	3–4 L of dialysis solution	90 min	Closed	Yes	0% postoperative	50% postoperative ND definition	ND
[25]	Yang et al.	2011	34	50	MMC	30 mg	6 L of saline	60–90 min	Open	ND	0% ND	14.7% ND period (serious adverse events)	11 months
					CDDP	120 mg							
[26]	Yang et al.	2010	30	50	HCPT	20 mg	12 L of saline	90 min	Open	ND	0% postoperative 30 days	14.3% ND period ND definition	9.6 months
					MMC	30 mg							
[60]	Glehen et al.	2010	150	53.4	MMC	30–50 mg/m^2^	ND	60 – 120 min	Closed/ Open	Yes	6.5% postoperative	27.8% postoperative (grade III–IV)	9.2 month
					CDDP	50–100 mg/m^2^							
					OX IR 5FU	360–460 mg/m^2^ 100–200 mg/m^2^ ND							
[34]	Scaringi et al.	2008	37	53,7	MMC	120 mg	12 L of saline	90–120 min	Open	Yes	5.4% 30 days	27% postoperative ND definition—complications listed	23.4 months
					CDDP	200/m^2^							
[37]	De Roover et al.	2006	16	ND	MMC	15 mg/m^2^	ND	73 min	ND	ND	6.25% postoperative	ND postoperative ND definition—complications listed	ND
[27]	Yonemura et al.	2005	107	52	MMC	30 mg	8 L of saline	ND	Open	ND	2.8% postoperative	21.5% postoperative ND definition—complications listed	11.5 months
					CDDP	300 mg							
					Etoposide	150 mg							
[28]	Hall et al.	2004	34	54,5	MMC	40 mg	4 L	120 min	Closed	ND	0% 30 days	35% ND period ND definition	8 months
[29]	Yonemura et al.	2001	48	ND	MMC	30 mg	8–10 L	60 min	Open	ND	4% ND	19% ND period (major operative complications)	ND
					CDDP	300 mg							
[30]	Fujimoto et al.	1999	71	58.5	MMC	~ 40 mg	3–4 L	120 min	Closed	Yes	0% ND	11,4% Postoperative ND period—complications listed	Survivals 2 year—88% 4 year—76% 8 year—62%
[31]	Fujimoto et al.	1997	48	Group 1–56.9 Group 2–48 Group 3–47.4	MMC	~ 40 mg	3–4 L of dialysis solution	120 min	Closed	Yes	ND	ND	ND
[32]	Yonemura et al.	1996	83	60	MMC	30 mg		60 min	Open	ND	1,2% ND	5,8% ND period ND definition—complications listed	46 months
					CDDP	120 mg							
					Etoposide	150 mg							
[64]	Hamazoe et al.	1994	42	56.5	MMC	10 μg/mL	8–12 L	50–60 min	Closed	ND	ND	ND	77 months
[65]	Fujimura et al.	1994	22	60.3	MMC	30 mg/kg	10 L	60 min	Open	ND	0% Postoperative	36% ND period morbid events associated to HIPEC—complications listed	Survivals:1 year—95% 2 year—89% 3 year—68%
					CDDP	300 mg/kg							
[33]	Yonemura et al.	1991	41	56	MMC	50 mg	8–10 L	40–60 min	Open	ND	NDND	ND	14.6 months
					CDDP	300 mg							

Papers studied on abstract base only, lacking some information; ND: not discussed; 5FU: 5-fluorouracil; CDDP: cisplatin; OX: oxaliplatin; MMC: mitomicyn C; SPC: synchronous peritoneal carcinomatosis; MPC: metachronous peritoneal carcinomatosis; IR: irinotecan; CRS: cytoreductive surgery; PCI: peritoneal carcinomatosis index, CHIP: chemotherapeutic hyperthermic intraperitoneal perfusion, HIPEC: hyperthermic intraperitoneal chemotherapy, DOC: docetaxel, LP: lobaplatin, LS-HIPEC: laparoscopic hyperthermic intraperitoneal chemotherapy, LDG: laparoscopy distal gastrectomy, i.v.: intravenous. 1 Authors divided the population of patients into subgroups.
